# Preterm Birth Is Correlated With Increased Oral Originated Microbiome in the Gut

**DOI:** 10.3389/fcimb.2021.579766

**Published:** 2021-06-17

**Authors:** Chunhua Yin, Jingrui Chen, Xuena Wu, Yeling Liu, Quan He, Ying Cao, Yi-E Huang, Sisun Liu

**Affiliations:** ^1^ Department of Obstetrics and Gynecology, The First Affiliated Hospital of Nanchang University, Nanchang, China; ^2^ Anatomy and Pathology Department, Jiangxi Health Vocational College, Nanchang, China; ^3^ Nursing Department, The First Affiliated Hospital of Nanchang University, Nanchang, China; ^4^ Shenzhen Baoan Women’s and Children’s Hospital, Jinan University, Shenzhen, China

**Keywords:** preterm birth, preterm birth subtypes, gut microbiome, maternal gut microbiome, bacteria translocation

## Abstract

**Background:**

Preterm birth is one of the leading causes of perinatal morbidity and mortality. Gut microbiome dysbiosis is closely related to adverse pregnancy outcomes. However, the role of the gut microbiome in the pathogenesis of preterm birth remains poorly studied.

**Method:**

We collected fecal samples from 41 women (cases presenting with threatened preterm labor =19, 11 of which delivered preterm; gestational age-matched no-labor controls, all of which delivered at term = 22) were recruited for the study. We performed 16S rRNA amplicon sequencing to compare the composition of the gut microbiome in threatened preterm labor cases and controls and among women who delivered preterm and at term. By annotating taxonomic biomarkers with the Human Oral Microbiome Database, we observed an increased abundance of potential oral-to-gut bacteria in preterm patients.

**Results:**

Patients with preterm birth showed a distinct gut microbiome dysbiosis compared with those who delivered at term. Opportunistic pathogens, particularly *Porphyromonas*, *Streptococcus*, *Fusobacterium*, and *Veillonella*, were enriched, whereas *Coprococcus* and *Gemmiger* were markedly depleted in the preterm group. Most of the enriched bacteria were annotated oral bacteria using the Human Oral Microbiome Database. These potential oral-to-gut bacteria were correlated with clinical parameters that reflected maternal and fetal status.

**Conclusions:**

This study suggests that patients who deliver preterm demonstrate altered gut microbiome that may contain higher common oral bacteria.

## Introduction

Preterm birth, defined as birth before 37 weeks, is one of the leading causes of global neonatal morbidity. An estimated 15 million infants are born preterm every year (Blencowe et al., 2012; Liu et al., 2016). Preterm birth complications can increase the risk of metabolic abnormalities, respiratory distress, and poor neurobehavioral development in mothers and newborns (Mwaniki et al., 2012; Platt, 2014). Although infectious and sterile inflammation, oxidative stress and maternal hormone imbalance are recognized as important factors for preterm birth (Romero et al., 2005; Keelan, 2018), the etiology remains controversial.

In the recent decade, growing evidence has shown that the human microbiome plays a critical role in the development of many diseases. While the changes in the maternal vaginal microbiome and the development of the infant microbiome across different body sites have been well studied, the relationship between the maternal gut microbiome and preterm birth is rarely reported (Mychaliska, 2014). The dysbiosis of the maternal gut microbiome could result in severe gestational disease, including preeclampsia, gestational diabetes mellitus and metabolic syndrome (Koren et al., 2012; Wang et al., 2018; Chen et al., 2020). The gut microbiome may deliver new insights into understanding mechanisms and potential clinical strategies to detect the risk of preterm delivery and prevent preterm birth.

In the present study, we recruited patients experiencing threatened preterm labor and healthy pregnant women not in labor to provide fecal samples at similar gestational age to investigate the association of intestinal microbes with the outcome of preterm birth. We performed 16S rRNA gene amplicon sequencing to identify gut bacteria exhibiting altered abundance in women who experienced early/late preterm birth.

## Methods

### Research Participants and Sample Collection

Healthy pregnant women and those with threatened preterm labor were recruited in the Department of Obstetrics, The First Affiliated Hospital of Nanchang University, Jiangxi Province, China. Ethical approval of the study was granted by the Ethics Committee of the First Affiliated Hospital of Nanchang University (2019-061). All participants have been informed of the purpose, background, process, risks, and benefits of the study, and signed the informed consent to participate in this study. The Department of Gynecology and Obstetrics, the First affiliated Hospital of Nanchang University was responsible for the study.

The inclusion criteria of patients: (1) The patients delivered between 28 weeks and 37 weeks of gestation, which were classified into preterm group. (2) The patients at 28 weeks and less than 37 weeks gestation suffered regular or irregular contractions accompanied by progressive dilation or shortening of the cervical canal, which were classified into Sym.preterm group in this study.

The exclusion criteria of patients: (1) Administration of any antibiotic or probiotic treatment one month before sample collection. (2) Diseases that may affect microbiome composition such as thyroid disorders, asthma, lipid metabolic disorders, inflammatory bowel disease, irritable bowel syndrome, and celiac disease. (3) Other obstetric conditions complicating pregnancy, such as gestational hypertension, gestational diabetes, twin or multiple pregnancies, placenta previa. (4) Other chronic diseases, such as chronic hypertension, chronic kidney disease. (5) Termination of pregnancy due to fetal or maternal factors. Fecal samples were collected from all enrolled subjects at the hospital, while samples from patients with threatened preterm labor were taken at symptom initiation. The fecal samples were collected at admission and the average sampling time of preterm group was 25.9 gestational weeks and 26.5 gestational weeks for the control group. All fecal samples were stored at −80°C until further processing.

### Bacterial Genomic DNA Extraction, Sample Processing, and Sequencing

Bacterial genomic DNA was extracted using a MinkaGene Stool DNA kit (Magigene, Guangdong, China) according to the manufacturer’s instructions. After extraction, the 16S rRNA V4 region was amplified by quantitative real-time PCR with the following barcoded primers (shown from 5′ to 3′): V4F, GTGYCAGCMGCCGCGGTAA and V4R, GGACTACNVGGGTWTCTAAT. Sun and colleagues (Sun et al., 2013) showed that the V4 and V5 regions of bacteria 16S rRNA concentrate the least intragenomic heterogeneity. Further works verified its efficiency by revision to the region primer and avoided its bias to particular taxa like Proteobacteria (Caporaso et al., 2011; Caporaso et al., 2012; Parada et al., 2016; Walters et al., 2016). Therefore, in this study, 16S rRNA V4 region was selected for sequencing. Briefly, amplifications were performed using a step cycling protocol consisting of 98°C for 30 s, 35 cycles at 98°C for 10 s, 54°C for 30 s, and 72°C for 45 s, ending with the final elongation at 72°C for 10 min. PCR products were purified using an AxyPrep PCR Cleanup Kit (Axygen, California, U.S.A.). Sequencing libraries were generated using TruSeq DNA PCR-Free Sample Preparation Kit (Illumina, USA) following the manufacturer’s recommendations, and index codes were added. The library quality was assessed on the Qubit@ 2.0 Fluorometer (Thermo Scientific) and Agilent Bioanalyzer 2100 system. The MiSeq platform (Illumina, 2 × 250 bp paired-end, CA, USA) was employed for the 16S rRNA sequencing.

### Bioinformatics Processing

QIIME2 was used for controlling the sequencing data quality and clustering the 16S rRNA gene reads into Amplicon Sequence Variants [ASVs, based on DADA2 pipeline (Bolyen et al., 2019)], taxonomic assignment (based on the Greengenes Database V.13_8), and performing alpha diversity (Observed ASVs, Shannon Index and Phylogenetic Diversity Whole Tree Index), beta diversity, and PCoA analyses. All samples were rarefied to 3,000 sequences, and three samples were filtered. Permutational multivariate analysis of variance (PERMANOVA) was performed to determine if the microbiota composition differed between groups and generate the explained variation based on distance metrics. Linear discriminant analysis effect size (LEfSe) was performed to identify the bacterial biomarkers between groups (Segata et al., 2011). Distance-based redundancy analysis and variance projection were employed to show the tendency of variables. Oral bacteria were annotated according to the Human Oral Microbiome Database (HOMD, www.homd.org) (Chen et al., 2010). Sequencing reads had been uploaded on European Nucleotide Archive (access number: PRJEB39133).

### Statistical Analysis

The significance of differences between the two groups was determined by the Wilcoxon rank-sum test. The Kruskal–Wallis test was used for multi-groups. P values less than or equal to 0.05 were considered significant. The Benjamini and Hochberg method was used to adjust the P-value for multiple hypotheses (Benjamini and Yekutieli, 2001). Correlation analyses were performed based on the Pearson’s product–moment correlation. Statistical analyses and data visualization were performed using R V.3.5.0 (under RStudio V.1.1.453), with the vegan, ggplot2, pheatmap and corrplot packages.

## Results

### Gut Microbiome Profiles Differ in Preterm Patients

After rarefied to 3,000 sequences, a total of 19 women with threatened preterm labor and 22 healthy pregnant women were included in the present study. In the threatened preterm group, eight patients with preterm symptoms delivered (Sym.PTB) at term, and 11 patients delivered preterm. The preterm-delivered group was further divided into an early week preterm group (seven women, Early PTB) and a late-week preterm group (four women, Late-PTB), depending on whether the condition was detected before 34 weeks of gestation. The clinical parameters are summarized in **Table 1**.

**Table 1:** Characteristics of the study cohort.

	Health (N = 22)	Preterm (N = 19)	Preterm subgroups		
			P.sym (N = 8)	P.late (N = 4)	P.early (N = 7)
Age	34.0 (5.30)	32.6 (4.54)	30.5 (3.63)	33.8 (5.50)	34.4 (4.54)
Gestational weeks	38.7 (1.19)	32.9 (7.45)	39.1 (1.37)	34.9 (0.443)	24.6 (5.43)
Weight	71.5 (7.32)	59.6 (8.91)	52.4 (7.14)	62.8 (1.89)	65.0 (8.47)
BMI at delivery	28.6 (2.25)	24.4 (3.98)	21.4 (3.21)	25.8 (1.49)	26.6 (3.97)
Neonatal weight	3,140 (368)	1,860 (1120)	3,000 (418)	2,360 (218)	756 (558)

**Figure 1:**
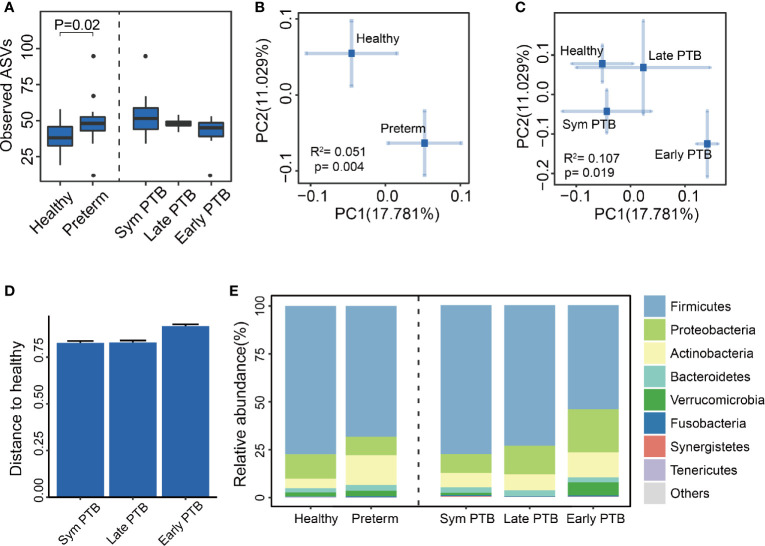
The diversity and composition of the gut microbiome. **(A)** Observed ASVs of all groups. Observed ASVs between the healthy group and the preterm group (Wilcoxon rank-sum test), along with the preterm symptoms delivered group (Sym PTB), late-week preterm group (Late PTB) and early week preterm group (Early PTB). (Subgroups are compared with healthy group using Wilcoxon rank-sum test and adjusted by the Benjamini and Hochberg method). **(B, C)** Bray–Curtis distances PCoA of all groups. PCoA of Bray–Curtis distances for the bacterial community structure of the gut microbiome between the healthy group and the preterm group **(B)**, the healthy group and the Sym PTB group and the late PTB group and the early PTB group. **(C)** The eigenvalues of axe PC1 and PC2 were 0.29 (17.781%) and 0.46 (11.029%), respectively. The eigenvalues of axe PC1 and PC2 were 0.60 (17.781%) and 0.97 (11.029%), respectively. PERMANOVA was employed. **(D)** Preterm subgroup distances to healthy group. The distance of the preterm birth subgroups to the healthy group, based on Bray–Curtis distances. **(E)** Relative abundance of all groups. Comparison of the relative abundance of the dominant phylum in the healthy group, the preterm group, and the preterm birth subgroups.

The fecal samples of these participants were collected for DNA extraction and 16S rRNA sequencing. By employing QIIME2 bioinformatic software, we showed that alpha-diversity indices, including observed ASVs, Shannon Index, and PD Whole Tree Index, were increased in the preterm group, indicating a higher richness and evenness in preterm patients than in healthy controls. We did not observe significant differences in alpha diversity within the preterm subgroups (**Figure 1A**, **Supplementary Figures S1A, B**).

Beta diversity was calculated using the Bray–Curtis distance metrics to measure the extent of similarity in fecal microbial communities. According to the principal co-ordinate analysis, the gut microbiome of the preterm group significantly differed from that of the healthy group (PERMANOVA, P = 0.004, R^2^ = 0.051, **Figure 1B**), indicating that the structure of the microbiota of preterm patients differed from that of healthy controls. Interestingly, we observed that the preterm subgroups showed a gradually drifting tendency away from the healthy groups with increasing severity (PERMANOVA, P = 0.019, R^2^ = 0.107, **Figures 1C, D**), which suggests there are distinct gut microbiome profiles among laboring women who experience term birth, late preterm birth, or early preterm birth.

Subsequently, we analyzed the phylum-level profiles for the gut microbiota between preterm patients and healthy controls, which were fairly similar. The dominant phyla of both healthy and preterm groups were Firmicutes, Proteobacteria, Actinobacteria, Bacteroidetes, Verrucomicrobia, and Fusobacteria (**Figure 1E**).

Taken together, these results demonstrated the presence of gut microbiota dysbiosis in preterm patients compared with healthy controls.

### Clinical Parameters Associated With Gut Microbiota Dysbiosis

Given that the gut microbiota was significantly different between preterm patients and healthy controls, we next investigated whether the gut microbiota was associated with the clinical parameters of patients.

To measure the extent of clinical parameters associated with the gut microbiome between preterm patients and healthy controls, we employed permutational multivariate analysis of variance to calculate the explained variation in host parameters based on the Bray–Curtis distance metric. Among 28 parameters with less than 20% missing data, five were significantly associated with gut microbial variations between preterm patients and healthy controls, namely, gestational age (recorded in weeks), neonatal weight, and Apgar scores (at 1, 5, and 10 min) (**Supplementary Figure S2A**). For the preterm subgroups, eight parameters were significantly associated with the subgroup microbiome variation, namely, C-reactive protein (CRP), tocolytic therapy, BMI at delivery, maternal age, gestational age, and Apgar scores (at 1, 5, and 10 min) (**Supplementary Figure S2B**). CRP is an inflammatory marker, while other parameters correlate with maternal–fetus prenatal and postpartum status, indicating that gut microbiome profiles are distinguished by gestational age at delivery and other clinical characteristics, many of which are expected to vary with gestational age at delivery.

**Figure 2:**
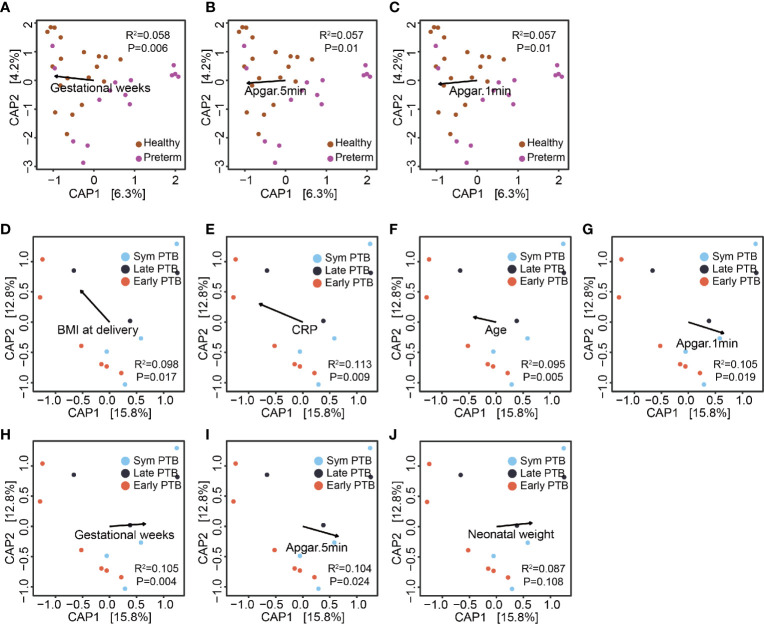
The association of the gut microbiome and host parameters. **(A–C)** Clinical features’ projection on all samples. The projection of continuous parameters in the healthy group and the preterm group samples, based on the Bray–Curtis distance metric, gestational weeks **(A)**, Apgar score at 5 min **(B)** and Apgar score at 1 min **(C)**, respectively. **(D–J)** Clinical features projection on preterm birth subgroups. The projection of continuous parameters in the preterm subgroup samples, based on the Bray–Curtis distance metric, BMI at delivery **(D)**, CRP **(E)**, age **(F)**; Apgar score at 1 min **(G)** gestational weeks (H); Apgar score at 5 min **(I)** and neonatal weight **(J)**, respectively.

We used distance-based redundancy analysis (db-RDA) to show the relationship of continuous parameters across samples by using R package vegan based on the Bray–Curtis distance metric. The projection of parameters demonstrated that gestational age (in weeks) and Apgar scores extended along axis-1, which distinguished between preterm patients and healthy controls (**Figures 2A–C**). While in the projection of preterm subgroup samples, CRP, BMI at delivery and maternal age were almost in the opposite direction to gestational age, neonatal weight, and Apgar scores, reflecting disease severity (**Figures 2D–J**). In the subgroup analysis, we included 13 preterm patients without missing value of all the included host parameters to avoid the projection change. Thus, the limited sample size could only provide us suggested indications, and further study on larger population is required. Altogether, these results again indicated that the gut microbiome profiles of preterm patients may be linked with other host parameters known to correlate with gestational age at birth.

### Potential Oral–Gut Bacterial Translocation in Preterm Patients

**Figure 3:**
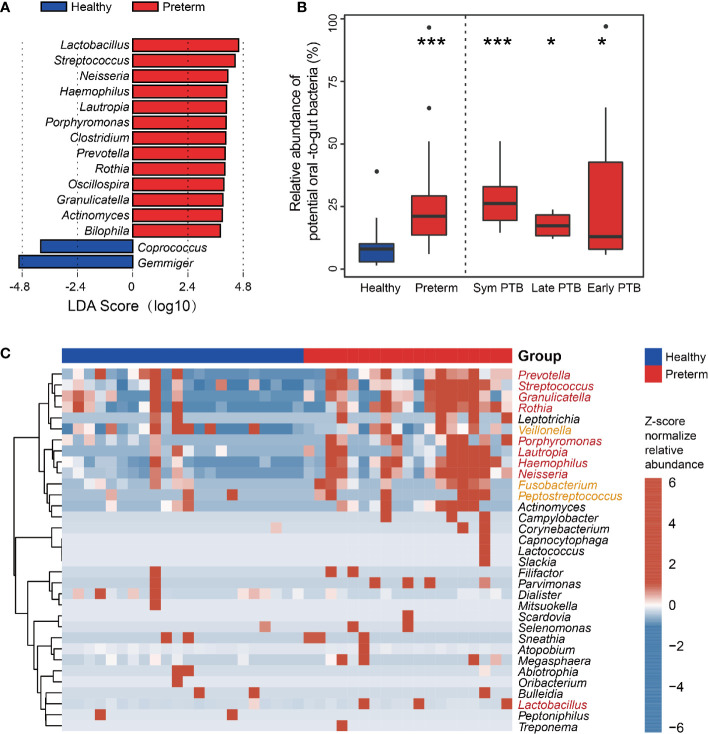
Identification of the gut microbial biomarkers between the healthy and preterm groups. **(A)** LEfSe analyses of healthy and preterm groups. Linear discriminant analysis effect size identified the genus between the healthy and preterm groups. Preterm-enriched taxa are indicated with a positive LDA score, and taxa enriched in healthy controls have a negative score. Only taxa meeting an LDA significant threshold of >3 are shown. **(B)** Total relative abundance of the common oral bacteria. The relative abundance of the common oral bacteria in the healthy group, the preterm group, and the preterm birth subgroups (healthy group and preterm group are compared using Wilcoxon rank-sum test; subgroups are compared with healthy group using Wilcoxon rank-sum test and adjusted by the Benjamini and Hochberg method). **(C)** Genus comparison between healthy and preterm group. Bacteria identified by the Wilcoxon rank-sum test with p < 0.05 are shown in the heatmap. The bar on the top indicates the group information of each sample. Red genus represents the common oral bacteria that overlapped with LEfSe results. Yellow genera represent the common oral bacteria but not overlapped with LEfSe results. *p < 0.05; ***p < 0.001.

To further identify the unique bacterial biomarkers between preterm and healthy groups, we performed LEfSe analysis based on the genus level and identified fifteen genera showing significant differences. Preterm patients exhibited a significant increase in the relative abundance of the genus *Fusobacterium, Streptococcus, Neisseria, Haemophilus, Lautropia, Porphyromonas, Clostridium, Prevotella, Rothia, Oscillospira, Granuliccatella, Actinomyces, and Bilophila*. On the contrary, genus Coprococcus and Gemmiger were depleted in preterm patients compared to those in healthy controls (**Figure 3A**).

Of note, several genera enriched in preterm groups were commonly considered oral pathogens, for example, Porphyromonas, Streptococcus, and *Fusobacterium*. As oral symptoms are common during pregnancy, we further examined the changes in the common resident oral bacteria in feces. We clustered and annotated bacteria to resident oral microbes according to the HOMD database. The relative abundance of common oral bacteria was markedly higher in the preterm group than in the healthy group (P < 0.001), as in the three subgroups (**Figure 3B**). We clustered the identified oral bacteria to examine their distribution among all individuals. Based on the heatmap, there was one cluster with thirteen genera enriched in preterm patients. Of these, nine were identified by LEfSe, and three were reported potential pathogens, indicating the possible higher intensity of bacterial oral–gut-translocation in preterm patients (**Figure 3C**). Among those enriched bacteria, Fusobacterium, Porphyromonas, and Streptococcus were common oral opportunistic pathogens.

Collectively, these results demonstrated that the gut microbiota of preterm patients differed from that of healthy individuals. Furthermore, a high proportion of genera enriched in preterm patients coincided with oral opportunistic pathogens.

### Potential Oral–Gut Translocated Bacteria for Distinguishing Women in Preterm From Healthy Control

**Figure 4:**
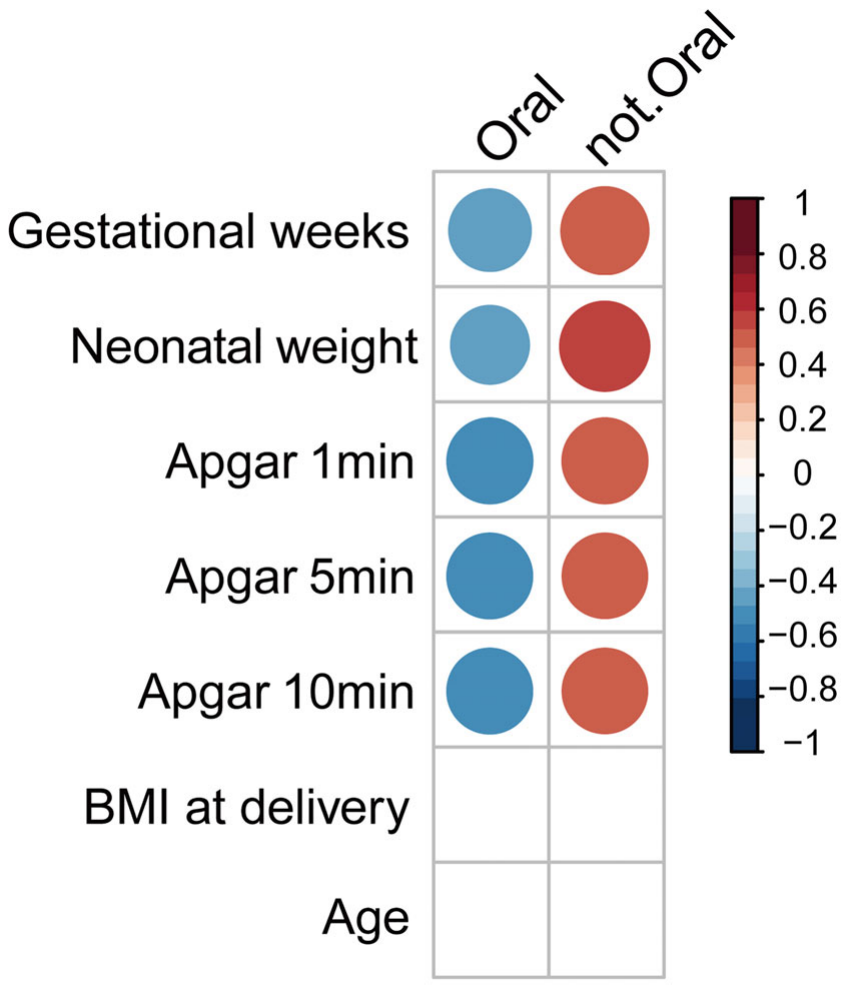
Correlations of parameters and abundance of oral-to-gut bacteria. Coefficient of correlation between clinical features and total potential oral bacteria. The relationship between the relative abundance of oral and non-oral bacteria and host parameters (gestational weeks; neonatal weight; Apgar scores in 1, 5, and 10 min; BMI at delivery).

Given that the gut microbiome profile was associated with the status of preterm patients and potential oral–gut translocated bacteria were enriched in preterm patients, we next investigated the association between potential oral–gut translocated bacteria and host parameters. We used Pearson’s product–moment correlation to evaluate the link between the relative abundance of oral-to-gut bacteria and host parameters. Results showed that oral-to-gut bacteria were negatively correlated with gestational age (in weeks), neonatal weight and Apgar scores (**Figure 4**). Altogether, the oral bacteria were correlated with clinical parameters reflecting the maternal and fetal status.

## Discussion

In the last few decades, a lack of effective methods for the prediction and prevention of preterm birth has placed a burden on the medical system and millions of families (Blencowe et al., 2012; Liu et al., 2016). The present study aimed to seek results from the microbiome. We found that maternal gut microbiome profiles were distinct in women with early *vs.* late preterm birth. The profile of the gut microbiome in preterm patients revealed that a group of common resident oral bacteria was enriched and associated with clinical parameters that reflected gestational and infantile status. These potential oral-to-gut bacteria, evaluated by the random forest model, would provide us another aspect to understand the relationship of gut bacteria and preterm birth.

The gut dysbiosis in preterm patients observed in our study echoed the findings of previous reports. Shiozaki et al. found compositional changes in the gut microbiome using terminal restriction fragment length polymorphism (Shiozaki et al., 2014). Interestingly, the gut dysbiosis pattern showed the enrichment of *Porphyromonas*, *Fusobacterium*, *Veillonella*, *Streptococcus*, *Bilophila*, and *Haemophilus* in preterm patients. Similar to the gut bacterial profile of pregnancy adverse outcome patients, *Fusobacterium*, *Streptococcus*, and *Veillonella* are associated with chronic inflammatory conditions, gut barrier damage, upregulate inflammation and led to adverse pregnancy outcomes (Chen et al., 2020). While in the present study, we found that alpha diversity increased in the preterm group. Although alpha diversity had been linked with gut health, it has also been reported to increase in different kinds of diseases, including gynecology and obstetrics disease (Prehn-Kristensen et al., 2018; Riquelme et al., 2019; Heshiki et al., 2020; Mrozinska et al., 2021). Thus, the dysbiosis pattern reported in this study is reliable.

Periodontitis is reported as a potential risk factor for preterm labor (Cobb et al., 2017; Chopra et al., 2020). *Porphyromonas*, commonly associated with periodontitis, can induce systemic inflammation, tissue damage, and possible maternal and fetal gut dysbiosis (Chopra et al., 2020). *Fusobacterium*, one of the opportunistic pathogens identified in the oral cavity and gut, can disrupt epithelial integrity and lead to tissue breakdown (Han, 2015; Yan et al., 2017). Recent studies showed that *Fusobacterium* was enriched in patients with adverse pregnancy outcomes and played a potential causal role in preeclampsia (Lv et al., 2019; Wang et al., 2019; Chen et al., 2020). In our study, *Porphyromonas* and *Fusobacterium*, which were common oral opportunistic pathogens, were significantly increased in the fecal samples of preterm patients. Though in the present study, we could not confirm whether these bacteria were originated in gut or translocated from oral cavity, the increase in relative abundance of these bacteria could lead to impairment of gut barrier (Chen et al., 2020)or oral epithelial barrier dysfunction (Fardini et al., 2011; Mahtout et al., 2011; Takeuchi et al., 2019). Yet the bacterial translocation from oral niche to the intestine is considered rare and aberrant. By studying salivary and fecal microbiome from several nations, researchers from Bork lab suggested that oral cavity could be an endogenous reservoir for gut microbiome, and oral–fecal transmission could play an important role in shaping the gut microbiome in health and disease (Schmidt et al., 2019). In our study, the enrichment of resident oral bacteria in the fecal samples of preterm patients could be the result of bacterial migration by swallowing through the digestive tract. Moreover, multiple studies indicated that dysbiotic gut bacteria could translocate to the placenta through impaired gut barrier (Chen et al., 2020). Although the existence of a placental microbiome is still debatable, there is a possibility that translocated pathogens could deteriorate placental structure and eventually lead to adverse outcomes (Aagaard et al., 2014; Seferovic et al., 2019). Our findings provide some evidence for the oral cavity being an endogenous reservoir for gut microbiome, potentially seeding it with oral microbes linked to preterm birth.

We also observed correlations between the oral bacteria and clinical parameters, such as gestational age, neonatal weight, and Apgar scores, indicating that the bacteria might reflect the disease status and outcomes. Our results suggested that the gut microbiota might have a potential to becoming a biomarker for distinguished preterm birth with further research on a larger population with rigorous control on variates like region and ethnicity (Deschasaux et al., 2018; He et al., 2018). The sample size in our study was relatively small and a larger population from multiple countries and races are required in future studies.

The present study has several limitations. First, for the sample and metadata collection, we only collected human fecal samples but missed oral sample like saliva or gem swab and the oral status of participants. Therefore, without clinical metadata on oral health and the oral microbiome sequencing data, we were unable to compare the similarity of gut and oral microbiome. As oral bacteria colonized in the gut are an unavoidable consequence and periodontitis is known etiologically linked to preterm birth through gut independent mechanisms, there are two ways to solve this problem in the future studies. One is employing meta-transcriptomics and shotgun metagenomics to evaluate the active transcription and cellular replication of oral strains in the fecal sample (Franzosa et al., 2014; Brown et al., 2016). And the second is performing animal experiment by constructing fluorescent opportunistic pathogen. Second, as the sample size was relatively small, our findings should be tested on repeated and larger population studies. Third, in the present study, the fecal samples from two out of nineteen patients in the preterm group were collected in labor. We could not rule out the effect of labor itself as a co-factor in this study. In future studies, collecting samples before labor or including a term labor control group would alleviate the effect of labor.

In summary, increased maternal oral bacteria in the guts of women experiencing preterm birth provided clues to further understanding the relationship between gut microbiome and preterm birth. However, the key role of microbiota in preterm birth pathogenesis and prospective mechanistic studies needs to be further investigated. Cohort studies that follow up from early pregnancy to *postpartum* and observe maternal gut microbiome dynamics along with fetal development will provide more comprehensive views on the effect of the gut microbiome on preterm birth. In conclusion, our study indicates that the gut microbiome in preterm birth women was significantly shifted compared with term women, which contained higher common oral bacteria.

## Data Availability Statement

The datasets presented in this study can be found in online repositories. The data presented in the study are deposited in the EBI repository (https://www.ebi.ac.uk/), accession number PRJEB39133.

## Ethics Statement

The studies involving human participants were reviewed and approved by the Committee of the First Affiliated Hospital of Nanchang University (2019-061). The patients/participants provided their written informed consent to participate in this study.

## Author Contributions

CY, JC and Y-EH designed the ideas and methods of this study; XW, JC and QH collected the data and processed the samples; JC, YL and YC analyzed the data; SL guided, supervised and supported the study. CY, JC and SL drafted and revised the manuscript. All authors contributed to the article and approved the submitted version.

## Funding

The study was financially supported by Science and technology research project of Jiangxi Education (No. GJJ170077 and No. GJJ181326) and Science and technology project of Health Commission of Jiangxi Province (No. 20201023).

## Conflict of Interest

The authors declare that the research was conducted in the absence of any commercial or financial relationships that could be construed as a potential conflict of interest.
