# Supplementary material for: Preterm Birth Is Correlated With Increased Oral Originated Microbiome in the Gut
Source: Front Cell Infect Microbiol. 2021 Jun 17;11:579766. doi: 10.3389/fcimb.2021.579766 (PMC8248533; doi:10.3389/fcimb.2021.579766)
Supplement: Supplementary file 1 [file DataSheet_1.docx]

**Supplementary Figures**

***Supplementary Material***


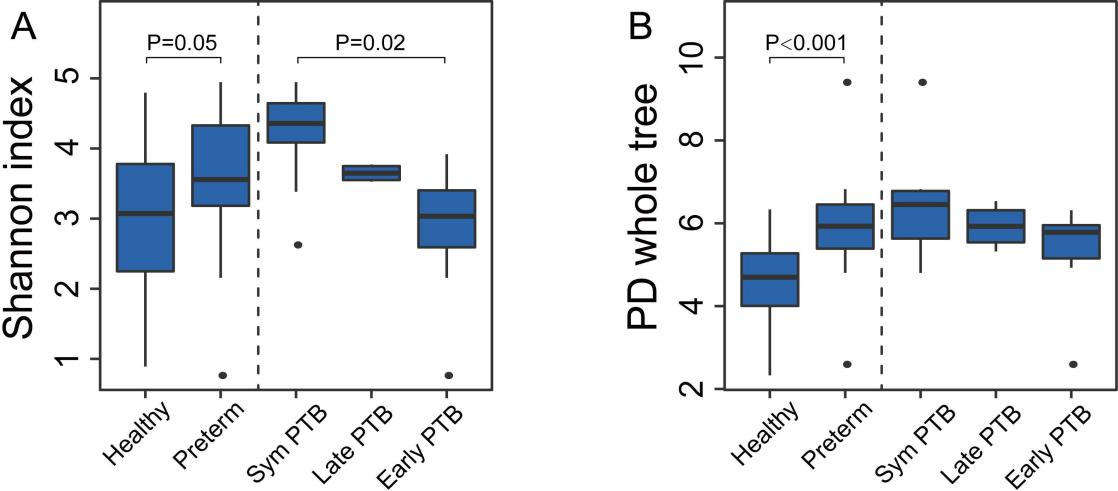


Figure S1 Alpha diversity of the Healthy group and the Preterm subgroups. (A) The Shannon Index between the Healthy group and the Preterm group (Wilcoxon rank-sum test), along with the Sym PTB group, the Late PTB group and the Early PTB group. (B) PD whole tree index between the Healthy group and the Preterm group (Wilcoxon rank-sum test), along with the Sym PTB group, the Late PTB group and the Early PTB group. Healthy group and preterm group are compared using Wilcoxon rank-sum test;Subgroups are compared with healthy group using Wilcoxon rank-sum test and adjusted by the Benjamini and Hochberg method.


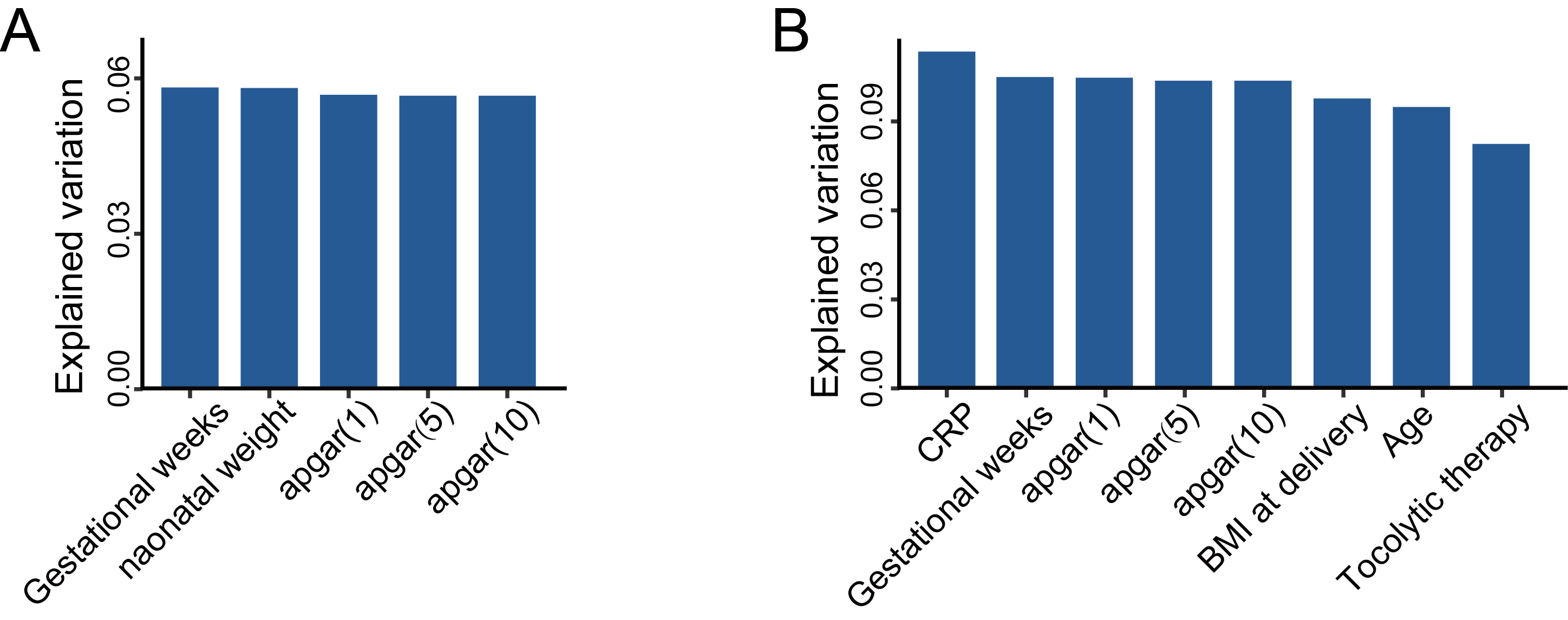


Figure S2 The association of the gut microbiome and host parameters. (A) The explained variations in the clinical characteristics between the Preterm group and the Healthy group (p < 0.05 in PERMANOVA). (B) The explained variations in the clinical characteristics among the Preterm subgroups (p < 0.05 in PERMANOVA)
